# Parasitization Activity by *Eretmocerus iulii* over the Orange Spiny Whitefly, *Aleurocanthus spiniferus*, in Sicily

**DOI:** 10.3390/insects16101074

**Published:** 2025-10-21

**Authors:** Alessia Farina, Carmelo Rapisarda

**Affiliations:** Applied Entomology Section, Department of Agriculture, Food and Environment (Di3A), University of Catania, 95123 Catania, Italy; carmelo.rapisarda@unict.it

**Keywords:** citrus, OSW, distribution, natural control, south Italy

## Abstract

**Simple Summary:**

The Orange spiny whitefly, an invasive insect first reported in Sicily in 2020, has become a serious problem for citrus production. From summer 2023, sporadic parasitization by a new parasitoid species, *Eretmocerus iulii*, was observed. Monitoring from late 2023 in three eastern Sicilian sites revealed that parasitization started very low (<1%), rose in autumn 2023 (under 10%), declined in winter, then increased sharply in spring 2024, peaking above 30% in May–June. Rates dropped during the hot summer but recovered in autumn 2024, reaching 15–20% higher than the previous year, and continued rising. Similar trends were recorded across sites. Growing parasitization consistently matched reduced whitefly nymph density, highlighting *E. iulii*’s potential as an effective, sustainable biocontrol agent for citrus.

**Abstract:**

Since its first report in 2020, *Aleurocanthus spiniferus* has rapidly spread in Sicily, causing alarm among citrus growers. To assess biocontrol possibilities, its spontaneous natural enemies were observed and, from summer 2023, sporadic parasitization was noted by an unknown species of the genus *Eretmocerus*, recently described as *E. iulii*. Parasitization over the OSW was studied regularly from November 2023 at two sites and from August 2024 at a third site (all of them in eastern Sicily). At the first two sites, parasitization was very low (<1%) at the beginning of the observations, increased during autumn 2023 (not exceeding 10%), decreased during winter 2023–2024 and rapidly increased again during spring 2024, peaking in May–June with over 30%. After declining during the hot and dry summer 2024, parasitization increased again in autumn 2024, reaching 15–20% higher values compared to the previous autumn, and continued to rise gradually until the end of the monitoring period. A similar parasitization trend was noted at the third site. At all three sites, increasing parasitization coincides with a lower density of OSW nymphs. These findings show the potential of *E. iulii* as a promising biological control agent, offering a sustainable solution to mitigate the effect of this whitefly on citrus production in Sicily.

## 1. Introduction

Whiteflies (Hemiptera: Aleyrodidae) are significant agricultural pests worldwide [1,2], which cause substantial economic losses to many crops, including vegetables (e.g., tomato or cucurbits), annual crops (e.g., cassava or cotton) and fruits (e.g., citrus) [3]. On these crops, whiteflies cause direct damage through sap-feeding and promotion of sooty-mould growth via honeydew excretion. Some whitefly species also cause serious indirect damage by vectoring devastating phytopathogenic viruses [4]. Many whiteflies are quarantine pests and impact international trade by hindering the export of plants and their products, thus posing a significant threat to agriculture.

Citrus production is severely impacted by whiteflies globally. Among the key whitefly species affecting citrus worldwide, the Citrus whitefly [*Dialeurodes citri* (Ashmead)], the Wooly whitefly [*Aleurothrixus floccosus* (Maskell)], the Australian citrus whitefly (*Orchamoplatus citri* Takahashi), and the two main citrus-feeding species of the genus *Aleurocanthus* Quaintance & Baker [namely the Citrus blackfly, *A. woglumi* Ashby, and the Orange spiny whitefly (OSW), *A. spiniferus* (Quaintance)] can be mentioned as the most harmful and widespread [5,6].

The whitefly genus *Aleurocanthus*, which still has an almost controversial taxonomy [7,8,9,10], presently includes more than 90 species, mostly occurring in tropical and subtropical regions and attacking cultivated plants, which makes these whiteflies important for quarantine in the EU [11].

Within this genus, *A. spiniferus* is the only species presently occurring in Europe. Native to South-East Asia and occasionally diffused throughout most of the tropical and subtropical regions of the world (especially in Africa, Australia, and Hawaii) [12], the OSW is one of the most harmful representatives of this whitefly genus, attacking a wide variety of plants and crops. It has approximately 90 host plant species [13,14,15,16], especially Rutaceae of the genus *Citrus* L.

It appeared in Europe less than two decades ago, when its presence was recorded in the Italian region of Apulia [17]. Over the following years, it was also found in Croatia, Montenegro, Greece, Albania, and France [14,15,18,19,20].

In Italy, after remaining limited to the region where it was initially found (Apulia) for about ten years, *A. spiniferus* spread rapidly over almost the entire country, including on plants other than citrus, and is presently known to occur in eleven out of the twenty Italian regions, from the North (Emilia-Romagna, Liguria, Lombardy), Centre (Latium, Marche, Tuscany) and South (Apulia, Basilicata, Calabria, Campania, Sicily) of the peninsula [12,16,17,21,22,23].

The feeding activity of *A. spiniferus* on citrus causes sooty mould development due to honeydew excretion, chlorosis, leaf drop, and the overall weakening of the plant, leading to significant economic losses in citrus production. Control measures for the whitefly are therefore needed.

Managing *A. spiniferus* requires a multifaceted approach, and integrated pest management (IPM) is needed [24] to prioritize biological control methods supplemented by selective chemical applications and cultural practices. Among the latter, pruning may have some importance in reducing OSW population pressure, by removing infested plant parts and enhancing airflow; the removal of alternative host plants in the citrus crop environment can also reduce pest reservoirs.

Chemical control, particularly based on applications with neonicotinoids (e.g., imidacloprid and acetamiprid), pyrethroids (such as deltamethrin and lambda-cyhalothrin) or insect growth regulators (like pyriproxyfen), has been largely used for OSW control, and is usually the most immediate measure applied against *A. spiniferus* infestations. Neonicotinoids can very easily reach feeding whiteflies on the undersides of leaves due to their systemic nature; however, their use has been recently restricted in the EU due to their impact on pollinators [25]. Although more respectful for beneficial insects, pyrethroids have demonstrated variable efficacy, often requiring repeated applications. Due to their selective action and lower non-target toxicity, insect growth regulators are better adapted in IPM programs for *A. spiniferus*: they target immature stages by disrupting moulting or mimicking juvenile hormones, leading to impaired development. For integrated control of the Orange spiny whitefly, plant derived insecticides (e.g., sweet orange essential oil, extracts of *Clitoria ternatea* L., or azadirachtin) also have an interesting use: they normally cause no significant direct mortality, but nevertheless induce behavioural changes (such as an impairment of both adult landing and oviposition) leading to reduced infesting performance in the pest insect [26]. Therefore, despite its widespread use, chemical control of *A. spiniferus* does not achieve satisfactory levels of effectiveness and does not ensure long-term control of the pest populations. Moreover, it raises additional concerns related to the possible development of resistance, which is highly frequent in whiteflies [27]; non-target effects on beneficial arthropods and residue issues in citrus products are also important challenges in the application of chemical control on whiteflies.

As for many citrus feeding whiteflies, biological control has a primary role in reducing populations of the Orange spiny whitefly. In its native areas, the insect is controlled by many natural enemies, including both predators, such as species from the orders Diptera, Neuroptera (lacewings), and Coleoptera (ladybirds) [21,28,29], and parasitoids, especially Hymenoptera Aphelinidae belonging to the genera *Encarsia* Förster and *Eretmocerus* Haldeman, but also *Amitus hesperidum* Silvestri (Hymenoptera Platygastridae) [28,30]. Successful biological control programs for the OSW have been carried out worldwide with *Encarsia perplexa* Huang & Polaszek [as *E. opulenta* (Silvestri)], *E. smithi* (Silvestri), and *A. hesperidum* [31,32,33,34,35], indicating the validity of these parasitoids as natural antagonists to be used in sustainable management of OSW infestations.

In Italy, a native predator from the family Coccinellidae [*Clitostetus arcuatus* (Rossi)] spontaneously adapted to prey on *A. spiniferus* [15]; in addition, two exotic ladybugs of the tribe Serangiini [*Delphastus catalinae* (Horn) and *Serangium montazerii* Fürsch] have been detected as active predators on OSW populations [15,36]. However, no significantly efficient parasitoids have been recovered in this country on *A. spiniferus* until very recently. In particular, surveys conducted during 2023–2024 in several Central and South Italian regions (including Sicily) found a new species of Aphelinid wasp, described as *Eretmocerus iulii* Laudonia and Melone, which proved to be an active and rapidly diffused parasitoid of the OSW [37], with parasitization rates ranging from almost 4% to over 70% based on the investigated regions and the sampling season [23]. During almost the same years, other parasitoid species appeared in different Italian regions (although not in Sicily so far), such as *Encarsia nipponica* Silvestri and *E. smithi* (Silvestri), probably as a result of spontaneous introductions [38,39].

Considering that Italy is among the ten leading citrus producing countries in the world and Sicily is the most important citrus producing region in Italy, OSW diffusion can pose a serious threat to the Sicilian citrus industry. For this reason, we carried out an investigation in this region aimed at assessing:

(i) the evolution of its population since the first findings in summer 2023, and
(ii) its potential role as a first step toward developing effective long-term biocontrol strategies against OSW in Sicily.

## 2. Materials and Methods

Monitoring activities were conducted monthly in three city centres located in the eastern part of Sicily (Siracusa, Caltagirone and Vizzini) from November 2023 to May 2025, although the start of observations varied among the survey sites. Measurements focused on ornamental *Citrus x aurantium* L., *Citrus limon* (L.), and *Citrus reticulata* (L.) (Table 1). The choice to conduct the investigations in urban settings and on ornamental citrus plants was driven by the need to obtain data in non-intensively cultivated environments, which are therefore not exposed to chemical insecticide applications.

During each sampling, thirty fully expanded leaves infested by *A. spiniferus* were collected from newly developed shoots, placed in hermetically sealed plastic bags, and transported to the laboratories of the Department of Agriculture, Food and Environment of the University of Catania, for detailed analyses using a stereomicroscope (Olympus Optical Co., Ltd., Tokyo, Japan, SZX-ILLK200).

### 2.1. Pest Abundance/Density

To obtain data on *A. spiniferus* abundance during the whole observation period, all healthy 4th instar nymphs of the whitefly were counted on each leaf (~45 cm^2^) at each sampling.

### 2.2. Parasitoid Parasitization Rate

The parasitization by *E. iulii* was evaluated only in terms of apparent parasitism rate, by counting the number of 4th instar nymphs of *A. spiniferus* having a parasitoid emergence hole in relation to the healthy 4th instar nymphs, following the same methodology described in the previous section and calculating the parasitism rate using the following formula:

$$\left[\frac{n{}^{\circ}\;of\;4th\;instar\;nymphs\;of\;A.\;spiniferus\;having\;E.\;iulii\;emergence\;holes}{total\;n{}^{\circ}\;of\;4th\;instar\;nymphs\;of\;A.\;spiniferus\;(healthy\;and\;with\;E.\;iulii\;emergence\;holes)}\right]*100$$

To verify that the emerging parasitoid was *E. iulii*, for each locality and each sampling, twenty healthy 4th instar nymphs of *A. spiniferus* were detached from leaves, and isolated in natural gelatin capsules (13.59 mm × 5.57 mm). They were stored at 25 ± 2 °C, 60 ± 5% R.H., with a photoperiod of 14L:10D h, until the emergence of the parasitoids, which were subsequently killed in 70% ethanol and subjected to morphological identification.

Specimens intended for this analysis were mounted on slides using a balsam-phenol medium [40], and the identification was carried out using a stereomicroscope (Olympus Optical Co., Ltd., Tokyo, Japan, SZX-ILLK200). Silvestri’s morphological description of *A. spiniferus* parasitoids [41] was consulted, in combination with identification keys provided by Compere [42], Gerling [43], and Hayat [44], and *E. iulii* morphological characters described by Laudonia et al. [37].

## 3. Results

The survey results showed that *A. spiniferus* was constantly present, along with *E. iulii*, on its major host plants, *Citrus* spp., during the entire monitoring period (November 2023–May 2025). The population trends of both insect species were similar across all three study localities.

### 3.1. Pest Abundance/Density

In all three analyzed locations, the observations carried out highlight how *A. spiniferus* exhibits a population density pattern influenced by seasonal variation, with higher numbers of nymphs recorded in winter 2023–2024 and summer 2024, followed by gradual declines during all subsequent months.

**Figure 1:**
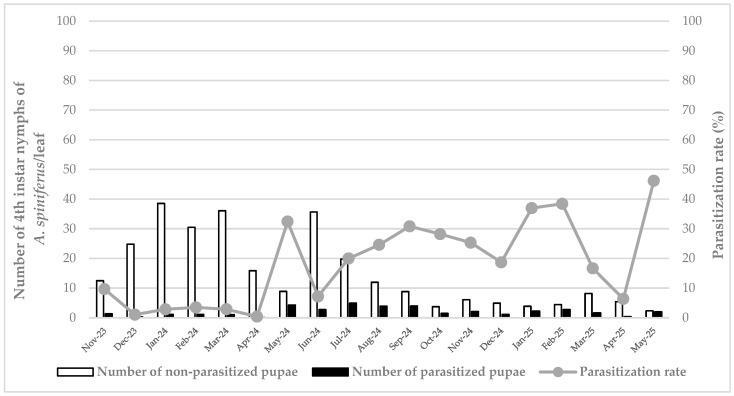
Density of healthy and parasitized (i.e., with an emergence hole) 4th instar nymphs of *Aleurocanthus spiniferus* and parasitization rate by *Eretmocerus iulii* in Siracusa, from November 2023 to May 2025.

Regarding the density of *A. spiniferus* in Siracusa, the data showed a high presence from December 2023 to March 2024, with the maximum value measured in January 2024 (0.86 individuals/cm^2^). In the following months, April–May 2024, the whitefly population decreased substantially (lowest value measured in May: 0.20 individuals/cm^2^), although it reached a new peak in June 2024 (0.79 individuals/cm^2^). Subsequently, from July 2024, the data show a gradual and almost constant decrease, with the lowest value measured in May 2025 (0.05 individuals/cm^2^) (Figure 1).

**Figure 2:**
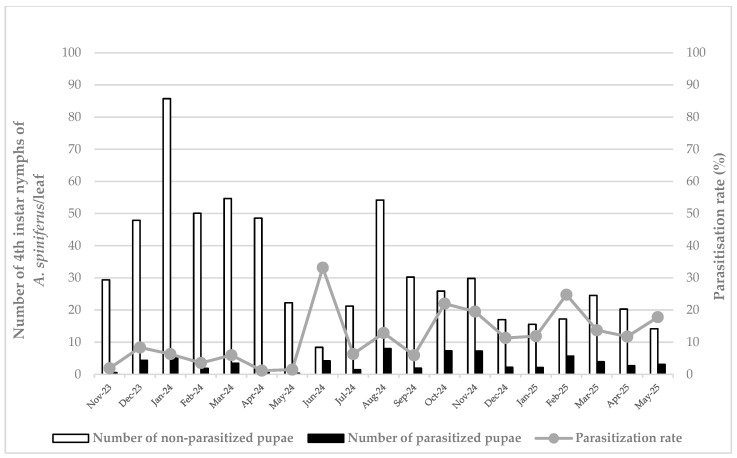
Density of healthy and parasitized (i.e., with an emergence hole) of 4th instar nymphs of *Aleurocanthus spiniferus* and parasitization rate by *Eretmocerus iulii* in Caltagirone, from November 2023 to May 2025.

As for Caltagirone, the results indicated a strong presence of *A. spiniferus* in December 2023 (1.91 individuals/cm^2^), with a subsequent phase from April to June 2024 in which the whitefly population decreased considerably (lowest value measured in June: 0.19 individuals/cm^2^). The pest density reached a peak again in August 2024 (1.20 individuals/cm^2^); but from September 2024 until the end of the monitoring activities, and almost the same as in Siracusa, the data showed a gradual and then stable decrease, with the lowest value measured in May 2025 (0.31 individuals/cm^2^) (Figure 2).

**Figure 3:**
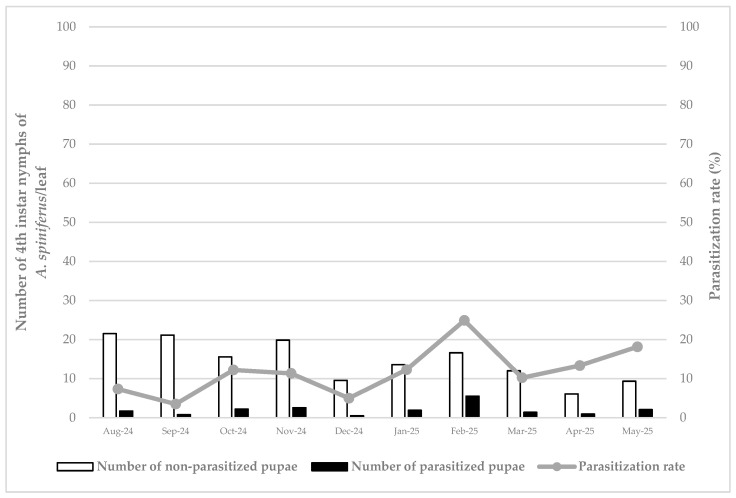
Density of healthy and parasitized (i.e., with an emergence hole) 4th instar nymphs of *Aleurocanthus spiniferus* and parasitization rate by *Eretmocerus iulii* in Vizzini, from August 2024 to May 2025.

Concerning the density of *A. spiniferus* in Vizzini, the data demonstrated a higher presence in August and September 2024 (0.48 and 0.47 individuals/cm^2^, respectively), with lower average values in October of the same year (0.35 individuals/cm^2^). A high number was recorded in November 2024 with the value of 0.44 individuals/cm^2^, but from December 2024 to May 2025, the population density declined and maintained a stable level (about 0.25 individuals/cm^2^) (Figure 3).

### 3.2. Parasitoid Parasitization Rate

Morphological analysis of the slide-mounted specimens enabled the identification of the parasitoid as *Eretmocerus iulii* Laudonia et Melone, very recently described [37].

A low initial parasitization rate by *E. iulii* was observed in Siracusa at the beginning of the monitoring activities, with the lowest value recorded in April 2024 (0.42%). The first peak in the parasitization rate was detected in May 2024 (32.41%), followed by low values (7.20%) observed in June 2024. From July 2024 to the end of the observations, the parasitization rate of *E. iulii* increased and remained approximately constant, with the maximum peak of 46.15% measured in May 2025, followed by 38.43% measured in February 2025 and 36.96% observed in January 2025 (Figure 1).

The parasitization rate at Caltagirone followed a similar trend as at the previous site, peaking in June 2024 (33.16%). Similarly, a low percentage was observed during the first months of the monitoring period, with the lowest value recorded in April 2024 (1.15%). From October 2024 to the end of the observations, the parasitization rate of *E. iulii* reached higher levels and approximately maintained an almost constant level, with another high peak of 24.78% detected in February 2025.

The parasitization rate at Vizzini showed a constant level (about 10–12%), with the highest peak reached, as at the previous sites, in February 2025 (24.89%), followed by 18.13% measured in May 2025 (Figure 3).

### 3.3. Pest Density and Parasitization Rate Relationship

**Figure 4:**
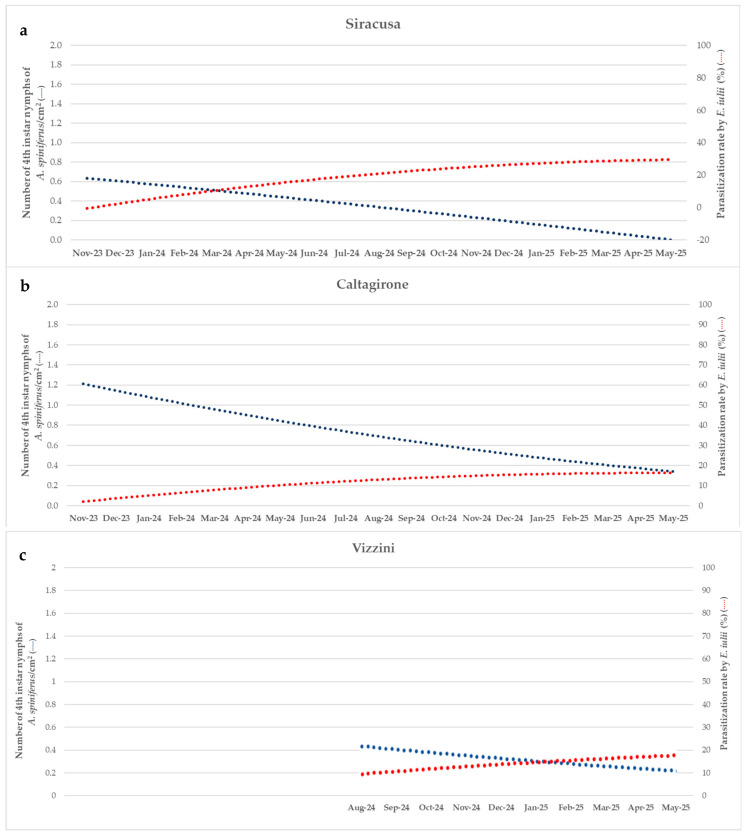
Temporal trend (November 2023–May 2025) of 4th instar *A. spiniferus* nymph density (blue line, left axis) and *E. iulii* parasitization rate (red line, right axis) in Siracusa (**a**), Caltagirone (**b**), and Vizzini (**c**). Trend lines highlight overall patterns and a potential inverse relationship between pest density and parasitization rate.

At the Siracusa site, a steady descending trend is observed in the number of *A. spiniferus* nymphs per cm^2^, which decreases consistently over the monitoring period. In contrast, the parasitization rate by *E. iulii* shows a clear rising trend, reaching its highest values toward the end of the study period (Figure 4a).

In Caltagirone, a clear decreasing trend was observed in the number of 4th instar nymphs of *A. spiniferus*/cm^2^; conversely, the parasitization rate by *E. iulii* showed a steady increase, rising from around 5% in November 2023 to nearly 17% by the end of the study period (Figure 4b).

In Vizzini, the density of 4th instar nymphs of *A. spiniferus* shows a progressive decline over time, decreasing from approximately 0.5 individuals/cm^2^ in August 2024 to 0.2 individuals/cm^2^ in May 2025. This trend suggests natural population suppression, potentially related to increasing parasitization activity by *E. iulii*, which starts around 10% and reaches nearly 20% by May 2025 (Figure 4c).

## 4. Discussion

The century-long history of biological control against *A. spiniferus* demonstrates that classical programs can yield long-term and cost-effective suppression of this pest. In the 1920s, severe outbreaks of *A. spiniferus* in Japan prompted classical biocontrol via *E. smithi*, which allowed rapid suppression of this introduced pest [45]. This established a template for later programs applied in the Pacific, when *E. smithi* was introduced artificially for controlling *A. spiniferus* in Guam (during the early 1950s), in Hawaii (in the 1970s), and in multiple islands of Micronesia (1980s–1990s) [46,47,48,49,50]. Later, in South Africa, after *A. spiniferus* arrived in the late 1980s, classical biocontrol with *E. smithi* delivered high parasitism (73–80%) within less than 8 months after release of the parasitoid, and sustained suppression of the whitefly in commercial citrus [51]. This proved to be cost-effective with a benefit ratio of 2.8 [32]. A successful biological control program has also been applied in Florida, with *E. perplexa* and *A. hesperidum* [35]. Thus, biological control, particularly through parasitoids, has a long history of success worldwide against the Orange spiny whitefly.

In Europe, the spontaneous arrival of various *Encarsia* (*nipponica*, *smithi*) and *Eretmocerus* (*iulii*) species indicates that natural enemy complexes are rebuilding in the areas newly invaded by the whitefly. The future perspective that these natural introductions, and the subsequent re-colonization by natural enemies, may have in the context of biological control of the Orange spiny whitefly give priority to actions including monitoring of the establishment of parasitoids, in parallel with preserving beneficial populations through selective management and evaluating possible augmentative release strategies. To this end, studies focusing on the spatio-temporal dynamics of the whitefly and its parasitoids emerging in different areas are needed, to better assess the potential of the newly introduced natural enemies to suppress the whitefly pest.

In the present study conducted in Sicily, the parasitization by *E. iulii* was extremely low initially (autumn 2023) in all surveyed sites, indicating a very recent spontaneous establishment of the natural enemy; in any case, it increased significantly starting from spring 2024 and has consolidated during the subsequent months of the investigation, demonstrating a quite stable presence of the parasitoid within the Sicilian environments under study.

Specifically, in Siracusa, the site located at the lowest altitude and characterized by the highest average temperatures among the three monitoring stations, *E. iulii* seems to have become more significantly established, providing a more effective natural control for the Orange spiny whitefly. In this location, the contrasting trends between the number of 4th instar nymphs of the whitefly and the parasitization rate (Figure 4a) clearly indicate a potential mutual negative correlation. This may reflect the validity of *E. iulii* as a natural enemy of *A. spiniferus*, which should be proposed as a good BCA for biological control programs for the whitefly.

In Caltagirone, the trend was similar as in Siracusa (Figure 4b), also revealing at this site the inverse correlation between pest density and parasitization rate, which indicates a potential causal relationship and supports the hypothesis that the parasitoid is contributing to the suppression of *A. spiniferus* in the area.

Also in Vizzini, on the other hand, the two trend lines reported in Figure 4c exhibit a clear inverse relationship between *A. spiniferus* density and *E. iulii* activity, supporting the hypothesis that a robust biological control effect is exerted by the parasitoid.

Pest density showed slight differences among the three sites, which may be related to their varying altitudes. The increases in *A. spiniferus* abundance observed during winter and summer, particularly in Siracusa and Caltagirone, could be associated with favourable climatic conditions during those periods. In contrast, the trend observed in Vizzini was more stable and less marked by distinctly high values. Remarkably, at the site in Siracusa, where parasitization by *E. iulii* remained relatively stable at higher levels (above 20%) for a longer period, starting as early as July 2024, and even reaching peak values close to 50%, the population of *A. spiniferus* showed the most significant reduction, with levels of presence approaching the near-total disappearance of the pest by the end of the investigation.

## 5. Conclusions

This study provides the first detailed evidence of the establishment and spread of *E. iulii* in Sicily and its role as a natural enemy of the invasive Orange spiny whitefly (*A. spiniferus*). Monitoring activities carried out from late 2023 to mid-2025 revealed that *E. iulii* has rapidly adapted to local citrus environments, progressively increasing its parasitization rates and showing a consistent inverse relationship with the pest’s density.

Although initial parasitization levels were very low, the parasitoid showed a clear capacity to expand and stabilize across different localities, with parasitization rates exceeding 40% in some areas. This trend suggests that *E. iulii* is not only persisting but also contributes significantly to the natural suppression of *A. spiniferus* populations. In Siracusa, where higher temperatures appear to favour its activity, pest densities declined most sharply, indicating that environmental factors may modulate the efficiency of natural control.

The findings confirm that *E. iulii* has the potential to become a valuable component of integrated pest management strategies in Sicily. Establishing sustainable regulation of *A. spiniferus* could reduce reliance on chemical control measures, limit the risks of developing resistance, and preserve beneficial insect communities in citrus orchards.

Future research should focus on long-term monitoring of *E. iulii* across broader agro-ecological contexts, possible interactions with other parasitoids and predators, and the evaluation of augmentative release programs to enhance its impact. Such efforts will be essential to consolidate the role of this newly described parasitoid in sustainable citrus pest management, not only in Sicily but potentially in other Mediterranean regions threatened by *A. spiniferus*.

## Figures and Tables

**Table 1:** Localities, host plants, and the start of monitoring activities of *Aleurocanthus spiniferus* and *Eretmocerus iulii* in Sicily conducted from November 2023 to May 2025.

Locality	Altitude	Coordinates	Host Plants	Starting Month
Siracusa (SR)	17 m a.s.l.	37.075136, 15.278588	*Citrus x aurantium* L. *Citrus limon* (L.)	November 2023
Caltagirone (CT)	608 m a.s.l.	37.237851, 14.513141	*Citrus x aurantium* L.	November 2023
Vizzini (CT)	586 m a.s.l.	37.164975, 14.752564	*Citrus x aurantium* L. *Citrus limon* (L.) *Citrus reticulata* (L.)	August 2024

## Data Availability

The original contributions presented in this study are included in the article. Further inquiries can be directed to the corresponding author.
